# Functional Outcomes After Holmium Laser Enucleation of the Prostate: Changes in the Urinary Flow Rate and Symptom Score During Short- and Mid-Term Follow-Up

**DOI:** 10.7759/cureus.107457

**Published:** 2026-04-21

**Authors:** Jorge Martin Millet, Viviana Farrera Gonzalez, Eduardo Cruz Nuricumbo, Hugo De La Rosa Barrera

**Affiliations:** 1 Urology, Hospital Regional de Alta Especialidad de la Peninsula de Yucatan (HRAEPY), Merida, MEX; 2 Endourology, Hospital Regional de Alta Especialidad de la Peninsula de Yucatan (HRAEPY), Merida, MEX

**Keywords:** benign prostatic hyperplasia (bph), holmium laser enucleation of the prostate (holep), international prostate symptom score (ipss), lower urinary tract symptoms, qmax

## Abstract

Objective: The objective of this study is to assess changes in the maximum urinary flow rate and symptom score in patients undergoing holmium laser enucleation of the prostate during short- and mid-term follow-up.

Materials and methods: This retrospective longitudinal study included patients treated with holmium laser enucleation of the prostate between January 2021 and December 2022 at a tertiary referral center. The urinary flow rate and symptom score were evaluated before surgery and at 1, 3, 6, 12, 18, and 24 months after the procedure. Comparisons between baseline and postoperative values were performed using appropriate statistical tests, with statistical significance set at p < 0.05.

Results: The analysis included 121 patients with a mean age of 69.9 ± 8.0 years. The mean urinary flow rate increased from 10.29 ± 5.27 mL/s before surgery to 20.01 ± 10.94 mL/s at one month, with continued improvement reaching 25.83 ± 10.70 mL/s at 24 months. The mean symptom score decreased from 20.29 ± 9.25 at baseline to 9.12 ± 6.66 at one month, with further reduction to 5.65 ± 4.16 at 12 months. The overall postoperative complication rate was 13.9%, and urinary incontinence decreased from 7.4% in the early postoperative period to 0.8% at 12 months.

Conclusions: In this cohort, holmium laser enucleation of the prostate was associated with sustained improvement in urinary flow and symptom burden, supporting its role as a durable surgical option for benign prostatic hyperplasia.

## Introduction

Benign prostatic hyperplasia is highly prevalent among aging men and represents one of the main causes of lower urinary tract symptoms, with a considerable impact on quality of life and daily functioning [1,2]. Symptom severity is commonly assessed using the International Prostate Symptom Score (IPSS), while the maximum urinary flow rate is widely used as an objective parameter to evaluate bladder outlet obstruction and response to treatment [2].

For many years, transurethral resection of the prostate was considered the standard surgical approach for benign prostatic hyperplasia. However, the introduction of holmium laser enucleation of the prostate has expanded surgical management by enabling complete anatomical removal of adenomatous tissue regardless of prostate size [3-6]. Due to its effectiveness, durability, and applicability across a broad range of prostate volumes, this technique has become an increasingly adopted surgical option [3-7].

Evidence from randomized trials, long-term studies, and meta-analyses indicates that holmium laser enucleation of the prostate is associated with significant improvements in urinary flow and symptom scores, with outcomes comparable or superior to those of transurethral resection of the prostate and other surgical techniques, while maintaining low retreatment rates [3-9]. More recent studies have also demonstrated favorable perioperative and short-term outcomes when compared with alternative approaches, including robot-assisted simple prostatectomy and other laser-based techniques [8].

Despite the growing body of international evidence, data from Latin American centers remain limited. Regional studies are particularly important in institutions adopting this technique during the learning curve, as outcomes may vary depending on surgical experience [3-7].

The objective of this study was to evaluate changes in the urinary flow rate and symptom score in patients undergoing holmium laser enucleation of the prostate during short- and mid-term follow-up at a tertiary referral center.

This work has not been previously presented at any scientific meeting or conference, nor has it been published in any form.

## Materials and methods

This was a retrospective longitudinal observational study conducted at a tertiary referral center (Hospital Regional de Alta Especialidad de la Peninsula de Yucatan (HRAEPY)). Institutional records were reviewed to identify patients who underwent holmium laser enucleation of the prostate between January 2021 and December 2022.

All consecutive patients treated during the study period were considered for inclusion. Demographic and clinical data were obtained from electronic medical records. Follow-up assessments were carried out according to routine clinical practice, and available information was collected at predefined postoperative intervals. No additional interventions or modifications to standard patient care were performed for the purposes of this study.

Eligible patients had a diagnosis of benign prostatic hyperplasia, documented preoperative urinary flow rate and symptom score values, and at least one postoperative follow-up evaluation. Patients with incomplete baseline information or insufficient follow-up for functional assessment were excluded.

The primary outcomes were the urinary flow rate and symptom score. Symptom severity was evaluated using the IPSS, a widely validated instrument for assessing lower urinary tract symptoms [1]. These variables were measured preoperatively and at one, three, six, 12, 18, and 24 months after surgery, depending on data availability. Baseline characteristics included age, prostate volume, and transition zone volume. Postoperative complications and urinary incontinence were also documented during follow-up.

Continuous variables are reported as mean ± standard deviation. Data distribution was assessed using the Shapiro-Wilk test. Comparisons between baseline and postoperative values were performed using either the paired t-test or the Wilcoxon signed-rank test, as appropriate. Baseline values were used as the reference for all postoperative comparisons. Longitudinal changes across follow-up were analyzed using the Friedman test. Statistical analyses were conducted using IBM SPSS Statistics for Windows, Version 26 (Released 2018; IBM Corp., Armonk, New York, United States), and a p-value < 0.05 was considered statistically significant.

## Results

The main functional analysis included 121 patients. The mean age was 69.9 ± 8.0 years, mean prostate volume was 77.4 ± 41.4 mL, and mean transition zone volume was 47.6 ± 23.3 mL. Baseline characteristics are presented in Table 1.

**Table 1:** Baseline patient characteristics Baseline demographic and clinical characteristics of patients undergoing HoLEP. Continuous variables are presented as mean ± standard deviation (SD). HoLEP: Holmium laser enucleation of the prostate; IPSS: International Prostate Symptom Score

Variable	n	Mean ± SD	Minimum	Maximum
Age (years)	121	69.9 ± 8.0	50	87
Prostate volume (mL)	112	77.4 ± 41.4	20.0	342.0
Transition zone volume (mL)	112	47.6 ± 23.3	4.0	118.1
Preoperative Qmax (mL/s)	121	10.29 ± 5.27	—	—
Preoperative IPSS	121	20.29 ± 9.25	—	—

The urinary flow rate showed a marked increase after surgery. The mean preoperative value was 10.29 ± 5.27 mL/s and increased to 20.01 ± 10.94 mL/s at one month, with improvement maintained during follow-up, reaching 25.83 ± 10.70 mL/s at 24 months. Changes in the urinary flow rate are detailed in Table 2.

**Table 2:** Evolution of Qmax after HoLEP Changes in the maximum urinary flow rate (Qmax) following HoLEP at different postoperative time points compared with baseline. Values are expressed as mean ± standard deviation (SD). The preoperative value was used as the reference for all comparisons, which explains the identical baseline values shown across time points. p-values were calculated using a paired t-test or the Wilcoxon signed-rank test, as appropriate. HoLEP: Holmium laser enucleation of the prostate

Comparison	Preoperative Qmax (mean ± SD)	Postoperative Qmax (mean ± SD)	p-value
Preoperative vs 1 month	10.29 ± 5.27	20.01 ± 10.94	< 0.001
Preoperative vs 3 months	10.29 ± 5.27	19.57 ± 11.13	< 0.001
Preoperative vs 6 months	10.29 ± 5.27	20.82 ± 9.88	< 0.001
Preoperative vs 12 months	10.29 ± 5.27	23.29 ± 8.67	< 0.001
Preoperative vs 18 months	10.29 ± 5.27	21.70 ± 10.37	< 0.001
Preoperative vs 24 months	10.29 ± 5.27	25.83 ± 10.70	< 0.001

The symptom score also demonstrated a clear postoperative reduction. The mean preoperative value was 20.29 ± 9.25 and decreased to 9.12 ± 6.66 at one month, with additional reductions observed at three months (7.97 ± 5.99), six months (7.07 ± 4.71), and twelve months (5.65 ± 4.16), with stable values at eighteen months (7.67 ± 7.37) and twenty-four months (6.89 ± 6.60). Changes in the symptom score are presented in Table 3.

**Table 3:** Evolution of IPSS after HoLEP Changes in the International Prostate Symptom Score (IPSS) following HoLEP at different postoperative time points compared with baseline. Values are expressed as mean ± standard deviation (SD). The preoperative value was used as the reference for all comparisons, which explains the identical baseline values shown across time points. p-values were calculated using a paired t-test or the Wilcoxon signed-rank test, as appropriate. HoLEP: Holmium laser enucleation of the prostate

Comparison	Preoperative IPSS (mean ± SD)	Postoperative IPSS (mean ± SD)	p-value
Preoperative vs 1 month	20.29 ± 9.25	9.12 ± 6.66	< 0.001
Preoperative vs 3 months	20.29 ± 9.25	7.97 ± 5.99	< 0.001
Preoperative vs 6 months	20.29 ± 9.25	7.07 ± 4.71	< 0.001
Preoperative vs 12 months	20.29 ± 9.25	5.65 ± 4.16	< 0.001
Preoperative vs 18 months	20.29 ± 9.25	7.67 ± 7.37	< 0.001
Preoperative vs 24 months	20.29 ± 9.25	6.89 ± 6.60	< 0.001

**Figure 1:**
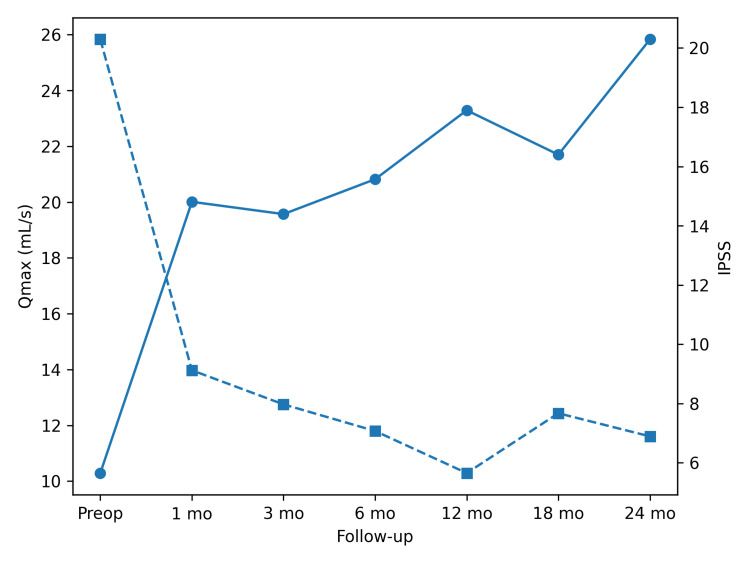
Postoperative changes in the urinary flow rate and symptom score The urinary flow rate increased after surgery and remained elevated throughout follow-up, while the symptom score decreased and remained low over time.

Longitudinally, both functional variables remained stable after the initial postoperative improvement. No statistically significant differences were observed among postoperative time points for either urinary flow rate (Friedman p = 0.423) or symptom score (Friedman p = 0.512), supporting sustained functional benefit over time. The combined postoperative trends of urinary flow rate and symptom score are illustrated in Figure 1.

Postoperative complications were evaluated in 122 patients with available follow-up records for this outcome. In total, 105 patients (86.1%) had no postoperative complications and 17 (13.9%) experienced at least one adverse event. The most frequent complications were urinary tract infection in eight patients (6.6%) and urethral stricture in six patients (4.9%), followed by overactive bladder symptoms in two (1.6%) and transient hematuria in one (0.8%). Postoperative complications are summarized in Table 4.

**Table 4:** Postoperative complications Postoperative complications following HoLEP. Data are presented as the number of patients (n) and percentage (%). HoLEP: Holmium laser enucleation of the prostate

Postoperative complications	n	%
No complications	105	86.1
Any complication	17	13.9
Urinary tract infection	8	6.6
Urethral stricture	6	4.9
Overactive bladder symptoms	2	1.6
Transient hematuria	1	0.8

Urinary incontinence was infrequent and showed progressive reduction over time. Among the 122 patients with available continence follow-up, urinary incontinence was present in nine patients (7.4%) at one week and in nine patients (7.4%) at one month, decreasing to five patients (4.1%) at six months and one patient (0.8%) at twelve months. The temporal evolution of urinary incontinence is shown in Table 5.

**Table 5:** Evolution of postoperative urinary incontinence Temporal changes in postoperative urinary incontinence following HoLEP. Data are presented as the number of patients (n) and percentage (%). p-values were calculated using a paired t-test or the Wilcoxon signed-rank test, as appropriate. HoLEP: Holmium laser enucleation of the prostate

Time point	Patients with urinary incontinence, n (%)	%	p-value
1 week	9/122	7.4	-
1 month	9/122	7.4	-
6 months	5/122	4.1	-
12 months	1/122	0.8	< 0.001

## Discussion

The findings of this study indicate that holmium laser enucleation of the prostate is associated with significant and sustained functional improvement in patients treated at a tertiary referral center. Both objective and subjective outcomes improved markedly after surgery, with urinary flow rate nearly doubling in the early postoperative period and symptom score showing a substantial reduction from baseline.

The improvement observed in the urinary flow rate is in line with prior randomized and long-term studies reporting effective relief of bladder outlet obstruction and sustained restoration of urinary flow following holmium laser enucleation of the prostate [3-7]. In this cohort, the mean urinary flow rate increased from 10.29 mL/s preoperatively to more than 20 mL/s as early as the first postoperative month and remained elevated throughout follow-up. These results are also consistent with systematic reviews and meta-analyses comparing this technique with transurethral resection of the prostate, where holmium laser enucleation has demonstrated durable efficacy and strong functional outcomes over time [9,10-13].

A comparable trend was observed in symptom burden following surgery. The mean symptom score decreased from the moderate-to-severe range at baseline to clearly lower postoperative values, with the most pronounced improvement during the first year and relative stability thereafter. This pattern is similar to that reported in long-term series and comparative studies involving other endoscopic approaches [4,9,11,12,14]. Taken together, the parallel improvement in the urinary flow rate and symptom score supports the functional effectiveness of this technique in routine clinical practice.

An additional observation was the stability of functional outcomes after the initial postoperative improvement. The absence of significant variation among later postoperative time points suggests that the benefit of this surgical approach is maintained during mid-term follow-up rather than being transient. This represents a key advantage of enucleation techniques and reinforces their role as durable, size-independent surgical options for benign prostatic hyperplasia [4-7,9].

The complication profile observed in this cohort was acceptable and comparable to that reported in previous studies. Most events were minor and managed conservatively. Postoperative urinary incontinence warrants particular attention because of its impact on quality of life [10]. Urinary tract infection and urethral stricture were the most frequent adverse events, both recognized complications after transurethral outlet surgery [11,12,15]. Recent clinical studies have also supported a favorable safety profile for this technique when compared with other minimally invasive or enucleation-based approaches [8].

In this cohort, urinary incontinence was uncommon and showed progressive improvement over time, consistent with previous series indicating that most cases are transient and resolve during follow-up [11,12,16-18]. This observation is particularly relevant during the learning curve, when surgical technique and mentorship may influence postoperative recovery. In this context, the findings are also aligned with recent data from Mexico reporting favorable short-term outcomes during structured training programs [19].

Several limitations should be considered. Due to its retrospective design, the study is subject to potential selection bias and does not allow causal inferences. In addition, variability in data availability across follow-up time points may have influenced the longitudinal assessment of outcomes. As the study was conducted at a single tertiary referral center, the generalizability of the findings to other clinical settings may be limited. The absence of a control group also precludes direct comparison with alternative surgical techniques, and residual confounding inherent to real-world clinical practice cannot be entirely excluded. Nevertheless, the study provides clinically meaningful real-world evidence from a Latin American tertiary referral center and supports the reproducibility of outcomes beyond high-volume international institutions. Recent surveys among urologists continue to highlight enucleation-based techniques as important surgical options for benign prostatic hyperplasia, reflecting an ongoing shift toward durable minimally invasive management [20,21].

## Conclusions

In this cohort, holmium laser enucleation of the prostate was shown to be a safe and effective surgical option for benign prostatic hyperplasia. The procedure was associated with sustained improvement in the urinary flow rate and symptom burden during mid-term follow-up, together with an acceptable complication profile and low rates of persistent urinary incontinence.

These results reinforce the value of this technique as a durable and reproducible option in routine clinical practice, particularly in tertiary referral centers and in settings where it is being adopted during the learning curve.

## References

[1] Barry MJ, Fowler FJ Jr, O'Leary MP (1992). The American Urological Association symptom index for benign prostatic hyperplasia. J Urol.

[2] Egan KB (2016). The epidemiology of benign prostatic hyperplasia associated with lower urinary tract symptoms: prevalence and incident rates. Urol Clin North Am.

[3] Infante Hernández S, Gómez Rivas J, Moreno Sierra J (2024). Benign prostatic hyperplasia. Med Clin (Barc).

[4] Elzayat EA, Elhilali MM (2006). Holmium laser enucleation of the prostate (HoLEP): the endourologic alternative to open prostatectomy. Eur Urol.

[5] Elzayat EA, Habib EI, Elhilali MM (2005). Holmium laser enucleation of the prostate: a size-independent new "gold standard". Urology.

[6] Gilling PJ, Fraundorfer MR (1998). Holmium laser prostatectomy: a technique in evolution. Curr Opin Urol.

[7] Ahyai SA, Lehrich K, Kuntz RM (2007). Holmium laser enucleation versus transurethral resection of the prostate: 3-year follow-up results of a randomized clinical trial. Eur Urol.

[8] Gilling PJ, Aho TF, Frampton CM, King CJ, Fraundorfer MR (2008). Holmium laser enucleation of the prostate: results at 6 years. Eur Urol.

[9] Grosso AA, Amparore D, Di Maida F (2024). Comparison of perioperative and short-terms outcomes of en-bloc Holmium laser enucleation of the prostate (HoLEP) and robot-assisted simple prostatectomy: a propensity-score matching analysis. Prostate Cancer Prostatic Dis.

[10] Shvero A, Calio B, Humphreys MR, Das AK (2021). HoLEP: the new gold standard for surgical treatment of benign prostatic hyperplasia. Can J Urol.

[11] Veronese N, Smith L, Pizzol D (2022). Urinary incontinence and quality of life: a longitudinal analysis from the English Longitudinal Study of Ageing. Maturitas.

[12] Elsaqa M, Zhang Y, Papaconstantinou H, Tayeb MM (2023). Incidence and predictors of urinary incontinence rates post-holmium laser enucleation of prostate. Low Urin Tract Symptoms.

[13] Das AK, Teplitsky S, Chandrasekar T, Perez T, Guo J, Leong JY, Shenot PJ (2020). Stress urinary incontinence post-holmium laser enucleation of the prostate: a single-surgeon experience. Int Braz J Urol.

[14] Sun F, Yao H, Bao X (2022). The efficacy and safety of HoLEP for benign prostatic hyperplasia with large volume: a systematic review and meta-analysis. Am J Mens Health.

[15] Chen F, Chen Y, Zou Y, Wang Y, Wu X, Chen M (2023). Comparison of holmium laser enucleation and transurethral resection of prostate in benign prostatic hyperplasia: a systematic review and meta-analysis. J Int Med Res.

[16] Wan Mokhter WM, Duan X, Yang J, Mohamed Daud MA (2025). Construction of a nomogram to predict urethral stricture after transurethral resection of the prostate: a retrospective cohort study. PLoS One.

[17] Coman RA, Bschleipfer T, Al Hajjar N, Petrut B (2024). Predictive factors of transient urinary incontinence following Holmium laser enucleation of the prostate (HoLEP): single-center experience. Medicina (Kaunas).

[18] Enikeev D, Taratkin M, Babaevskaya D (2022). Randomized prospective trial of the severity of irritative symptoms after HoLEP vs ThuFLEP. World J Urol.

[19] Aybal HC, Yilmaz M, Barlas IS, Duvarci M, Tuncel A, Tunc L (2024). Comparison of HoLEP, ThuLEP and ThuFLEP in the treatment of benign prostatic obstruction: a propensity score-matched analysis. World J Urol.

[20] Martinez-Salas AJ, Garcia-Rivera OU, Reyna-Blanco I, Jimenez-Garcia AD, Rosas-Hernandez H (2023). Adequate mentorship program for holmium laser enucleation of the prostate (HoLEP) leads to satisfactory short-term outcomes in the early learning curve of young urologists: first-year outcomes of a newly established mentorship training in Mexico. Cureus.

[21] Colvin H, Johnston M, Ripa F (2024). Transurethral resection and other minimally invasive treatment options for BPH: would we treat ourselves as we treat our patients? Results from EAU Section of Uro-Technology (ESUT) decision-making survey among urologists. Cent European J Urol.

